# Seasonal Differences in Relative Gene Expression of Putative Central Appetite Regulators in Arctic Charr (*Salvelinus alpinus*) Do Not Reflect Its Annual Feeding Cycle

**DOI:** 10.1371/journal.pone.0138857

**Published:** 2015-09-30

**Authors:** Anja Striberny, Chandra Sekhar Ravuri, Malcolm Jobling, Even Hjalmar Jørgensen

**Affiliations:** Department of Arctic and Marine Biology, UiT—The Arctic University of Norway, Tromsø, Norway; Centre of Marine Sciences & University of Algarve, PORTUGAL

## Abstract

The highly seasonal anadromous Arctic charr (*Salvelinus alpinus*) was used to investigate the possible involvement of altered gene expression of brain neuropeptides in seasonal appetite regulation. Pro-opiomelanocortin (*POMCA1*, *POMCA2*), Cocaine and amphetamine regulated transcript (*CART*), Agouti related Peptide (*AgRP*), Neuropeptide Y (*NPY*) and Melanocortin Receptor 4 (*MC4-R*) genes were examined. The function of centrally expressed Leptin (Lep) in fish remains unclear, so Lep (*LepA1*, *LepA2*) and Leptin Receptor (*LepR*) genes were included in the investigation. In a ten months study gene expression was analysed in hypothalamus, mesencephalon and telencephalon of immature charr held under natural photoperiod (69°38’N) and ambient temperature and given excess feed. From April to the beginning of June the charr did not feed and lost weight, during July and August they were feeding and had a marked increase in weight and condition factor, and from November until the end of the study the charr lost appetite and decreased in weight and condition factor. Brain compartments were sampled from non-feeding charr (May), feeding charr (July), and non-feeding charr (January). Reverse transcription real-time quantitative PCR revealed temporal patterns of gene expression that differed across brain compartments. The non-feeding charr (May, January) had a lower expression of the anorexigenic *LepA1*, *MC4-R* and *LepR* in hypothalamus and a higher expression of the orexigenic *NPY* and *AgRP* in mesencephalon, than the feeding charr (July). In the telencephalon, *LepR* was more highly expressed in January and May than in July. These results do not indicate that changes in central gene expression of the neuropeptides investigated here directly induce seasonal changes in feeding in Arctic charr.

## Introduction

In mammals, appetite is regulated by feedback mechanisms involving peripheral energy status, metabolic signals and centrally produced appetite regulators. Key to central regulation are two sets of neurons located in the arcuate nucleus (ARC) of the hypothalamus; appetite stimulating (orexigenic) neurons expressing Neuropeptide Y (NPY) and Agouti related peptide (AgRP), and appetite inhibiting (anorexigenic) neurons expressing Pro-opiomelanocortin (POMC) and Cocaine and amphetamine regulated transcript (CART). These exert their effects via second-order neurons expressing members of the NPY and Melanocortin (MC) receptor families [1, 2]. A major signalling molecule is the 16 kDa peptide Leptin (Lep) [3], which circulates proportionally to the amount of body fat in mammals and enters the ARC [4]. The Lep signal received by the Leptin receptor (LepR) is transduced via the Janus kinase (JAK) and signal transducer and activator of transcription (STAT) signalling pathway [5]. This leads to an activation of *POMC* and *CART* expression and an inhibition of *AgRP* and *NPY* expression [2].

Genes encoding many of the signalling molecules and their receptors found in mammals have been identified in fish [6] but evidence for appetite regulating roles in fish is inconclusive. For example, treatment of fish with NPY has consistently resulted in increased food intake [7–14], but feed deprivation studies have resulted in both an increase [13, 15–18] and no change [19–21] in brain *NPY* mRNA expression. There is support for the notion that Lep has an anorexigenic function in fish [22, 23], but there are currently no data that have revealed a lipostatic role of Lep in long-term energy regulation in fish [24–29]. In contrast to mammals, fish *Lep* is expressed in a wide range of tissues, including the brain [28, 30–33]. A possible role of centrally produced Lep in the regulation of appetite and energy homeostasis in fish has, however, received little attention. Most studies on central expression of appetite-regulating genes in fish have focused on either the hypothalamus or the whole brain, whereas the telencephalon and mesencephalon have been studied to a lesser degree [6].

Arctic charr (*Salvelinus alpinus*) occur in oligotrophic waters of the northern hemisphere [34] and in the northernmost part of their distribution range they can be anadromous and migrate to the sea each summer to feed [34, 35]. Marine prey can account for up to 90% of the annual food intake of the anadromous charr [36] and feeding is reduced or absent during overwintering in fresh water [37–40]. Furthermore, offspring of anadromous charr held in captivity at constant temperature fast voluntarily for several months during winter even though feed is available, implying that their seasonality is endogenously regulated [41]. As such, the anadromous charr can serve as a model for investigating mechanisms associated with long-term regulation of appetite and energy homeostasis in teleosts. A previous study with anadromous Arctic charr revealed no link between plasma Lep and changes in appetite and body adiposity across seasons [25]. To date it has not been investigated whether central Lep and LepR play a role in seasonal appetite regulation in the charr.

We hypothesised that the seasonal feeding cycle of anadromous Arctic charr is orchestrated by changes in gene expression of appetite regulators located in the brain and that differences in central expression of Lep and its receptor may play a role in long-term appetite regulation in Arctic charr. To this end, we investigated the gene expression of putative appetite regulators, including *Lep* and its signal transducing receptor *LepR*, in the hypothalamus, mesencephalon, and telencephalon of anadromous Arctic charr sampled during their natural seasonal feeding cycle in May (non-feeding), July (feeding) and January (non-feeding).

## Material and Methods

### Experimental set-up

The experiment used offspring of wild anadromous Arctic charr supplied by a government-run restocking program in the Hals watercourse (70°N), Finnmark, northern Norway. In the hatchery, the eggs were incubated in darkness at 6–8°C, and after hatching in April 2010 the fish were held at elevated temperature (∼10°C) and exposed to continuous light until July. From then on they were kept at ambient water temperature and fed in excess on commercial dry-pellet feed (Skretting, Stavanger, Norway) under continuous light until September 9, after which they were held on a 10:14-h light:dark cycle and at ambient water temperature until the start of the experiment. All feeding was by automatic feeders continuously for 24 h.

On March 6, 2012, a total of 420 individually tagged (passive integrated transponders) charr were transported from the rearing facility in Finnmark to the Aquaculture Research Station in Tromsø (ARST; 69°N). At ARST they were held in a 7 m^2^ circular tank supplied with fresh water at ambient temperature under natural light conditions (room with transparent roof) and fed in excess with commercial dry-pellet feed (Skretting, Stavanger, Norway) until April 24, when they were anaesthetized (benzocaine, 60 ppm) and measured for body mass and fork length to the nearest milligram and millimetre, respectively. Thereafter, 152 size-sorted fish with an average weight and length of 154.6 ± 1.6 g and 26.3 ± 0.1 cm, respectively, were distributed among two, 500 L circular tanks supplied with fresh water (76 fish per tank). The fish were then held under simulated natural light conditions (69°N) and fed in excess with commercial dry-pellet feed until the end of the experiment in January 2013.

Every two to four weeks from April 2012 to January 2013, all fish in both tanks were anesthetized in 60 ppm benzocaine, identified by tag reading and fork length and body mass measured. Periods of negative and positive weight change were considered to reflect non-feeding and feeding states, respectively. Sampling of fish for Reverse Transcription real-time quantitative PCR (RT-qPCR) was carried out between 10.00 am and 2.00 pm on three occasions: on May 15, July 27 (2012) and January 18 (2013). Five fish from each tank (total 10 fish per sampling time point) were killed by an overdose of benzocaine (150 ppm), brains were dissected out and the hypothalamus, mesencephalon, and telencephalon were immediately put separately in 1.5 ml Eppendorf tubes (Sigma-Aldrich Co. LLC, Switzerland) containing 1 ml RNAlater. Samples were kept at 4°C for ca. 24 h, and they were then stored at -20°C until extraction of total RNA. The sex of each fish was noted, the gonad removed and weighed, and when present, stomach contents were removed and weighed. As expected from the temporal change in growth rate of the fish groups, all fish sampled in May and January had empty stomachs, whereas those sampled in July had food in their stomach at the time of sampling (Table 1). Body condition of the fish sampled for gene expression studies was assessed using Fulton’s condition factor *K* calculated according to Ricker [42]: *K* = (*W* × *L* ^-3^) × 100, where *W* is body mass in g, and *L* is fork length in cm. Gonadosomatic index (*GSI*) was calculated as (*GW* × *W* ^-1^) × 100, where *GW* is the weight (g) of the gonad. All data are presented as mean ± standard error of mean (s.e.m.).

**Table 1:** Stomach content in % of body mass (BM), and gonadosomatic index (*GSI*) in % of BM of anadromous Arctic charr (*n* = 10) sampled during study.

Sampling Date	Stomach content [%BM ± s.e.m.]	*GSI* [%BM ± s.e.m.]
15.05.2012	0.0 ± 0.0	0.2 ± 0.1
27.07.2012	3.5 ± 0.4	0.1 ± 0.1
18.01.2013	0.0 ± 0.0	0.6 ± 0.1

The experiment was approved by the Norwegian Committee of Ethics in Animal Experimentation, Id no. 4187.

### Extraction of mRNA and RT-qPCR analyses

Tissues were disrupted using TissueLyser II (QIAGEN, Hilden, Germany), and RNA was extracted using the RNeasy Plus Universal Mini Kit (QIAGEN, Hilden, Germany) according to the manufacturer`s protocol. Concentration and purity of RNA were measured using NanoDrop ND2000c (Thermo Scientific, MA USA) and when the 260/280 or 260/230 absorbance ratio was below the quality threshold (1.7), samples were further purified using ethanol precipitation. Genomic DNA was removed by treating the RNA with Ambion TURBO DNA-free™ Kit (Life Technologies, CA, USA). A total of 2000 ng RNA was then reverse transcribed to cDNA using iScript™ Advanced cDNA Synthesis Kit for RT-qPCR (Bio-Rad, CA, USA). Reverse transcription was conducted according to the manufacturer`s protocol in a total reaction volume of 20 μl. No-reverse transcriptase (no-RT) controls were included in the reverse transcription step. The cDNA was diluted ten-fold. Amplification using qPCR was performed in duplicate using Hard-Shell® Low-Profile Thin-Wall 96-Well Skirted PCR Plates (Bio-Rad, CA, USA), 10 μl 2x SsoAdvanced™ Universal SYBR® Green Supermix (Bio-Rad, CA, USA), 1 μl primer mix (final concentration 250 nM) and 6 μl cDNA in a total reaction volume of 20 μl. Gene specific primers were designed by PrimerDesign (Southampton, UK) and verified for efficiency by performing RT-qPCR on serial dilutions (Table 2). Both no-RT controls, and one no-template control were included in the amplification step for each target gene. The amplification steps were as follows: 50°C for 10 minutes, 95°C for five minutes, [95°C for 10 seconds, 60°C for 30 seconds, plate read] x 40, 95°C for 10 seconds. The PCR product was subjected to a melt curve analysis with a temperature range of 65°C to 95°C, an increment of 0.5°C, and one plate read after each increment, to ensure product specificity. All qPCR analyses were run with CFX96 Real-Time PCR Detection System (Bio-Rad, CA, USA) and the software CFX Manager 3.0 (Bio-Rad, CA, USA).

**Table 2:** Forward (F) and reverse (R) primer sequences used for cDNA amplification by qPCR.

Gene		Primer Sequence (5’-3’)	bp	Accession number	Efficiency [%]
*Elongation factor 1 alpha (EF 1α)*	**F**	AGGCATTGACAAGAGAACCATT	119	AF498320.1	99.9
	**R**	TGATACCACGCTCCCTCTC			
*Pro-opiomelanocortin (POMC) A 1*	**F**	ACTGTTCAAAAATGTCATCATCAAAG	83	AB462418	106.4
	**R**	CACCTATCCTCCCTTCCTCTC			
*POMC A2*	**F**	GTTGGAGGAAAGAAGAGAGAGAA	119	AB462420	106.9
	**R**	CAATAACCACGCAGGACACA			
*Cocaine and amphetamine regulated transcript* (*CART*)	**F**	GTCCATCGTTCTTAGTGCTGAA	115	AB455538	107.1
	**R**	CAGTTGCTTTTCGTTGGTCAA			
*Melanocortin receptor 4* (*MC4-R*)	**F**	TTCTCACACTGGGGATAGTCA	113	AY534915.1	106.7
	**R**	CACAGCCAAAGAACAGATGAAT			
*Leptin* (*Lep*) *A1*	**F**	TCCTAGACTGGGCAGACCT	92	JQ615967.2	108.6
	**R**	GCCTGGGCAGCGTGATAT			
*LepA2*	**F**	TGGCACTAAACAGACTCAAGG	102	AB490667.1	92.3
	**R**	CTCAGTGATGATCTATGTCAGTAAC			
*Leptin receptor* (*LepR*)	**F**	CTTTGCTCGGGAGTCAGGA	129	KC683373.1	105.3
	**R**	CCTGTGCTTTGAGTGGACTG			
*Agouti related peptide* (*AgRP*)	**F**	TCGCCGAAGACCTGAAGAG	123	CA343080.1	104
	**R**	CGTGGTGCTGTCCCTGAT			
*Neuropeptide* (*NP*) *Y*	**F**	AGAATTGCTGCTGAAGGAGAG	83	AF203902	107.2
	**R**	GGGACAGACACTATTACCACAA			

All primers were produced and verified by PrimerDesign Ltd (Southampton, UK). Bp = product length in base pairs. Efficiencies were tested using serial dilutions.

### Data treatment and statistics

Relative fold change of gene expression was calculated using the *Δ*ΔC_t_ method [43]. Elongation factor 1 alpha (EF1α), a stable reference gene in Atlantic salmon (*Salmo salar*) [44], was used to normalise the C_t_ values of the target genes. Normalized qPCR data were LOG transformed prior to statistical testing [45].

A one-way ANOVA was used to test for differences in gene expression across the sampling dates.

The significance level was set to p < 0.05. When differences were found, post-hoc testing was carried out using Tukey’s Honestly Significance test. All statistical testing was done with SYSTAT 13 and figures were drawn using SigmaPlot 12.5 (both Systat Software, CA, USA).

## Results

### Seasonal changes in body mass and condition factor

**Fig 1:**
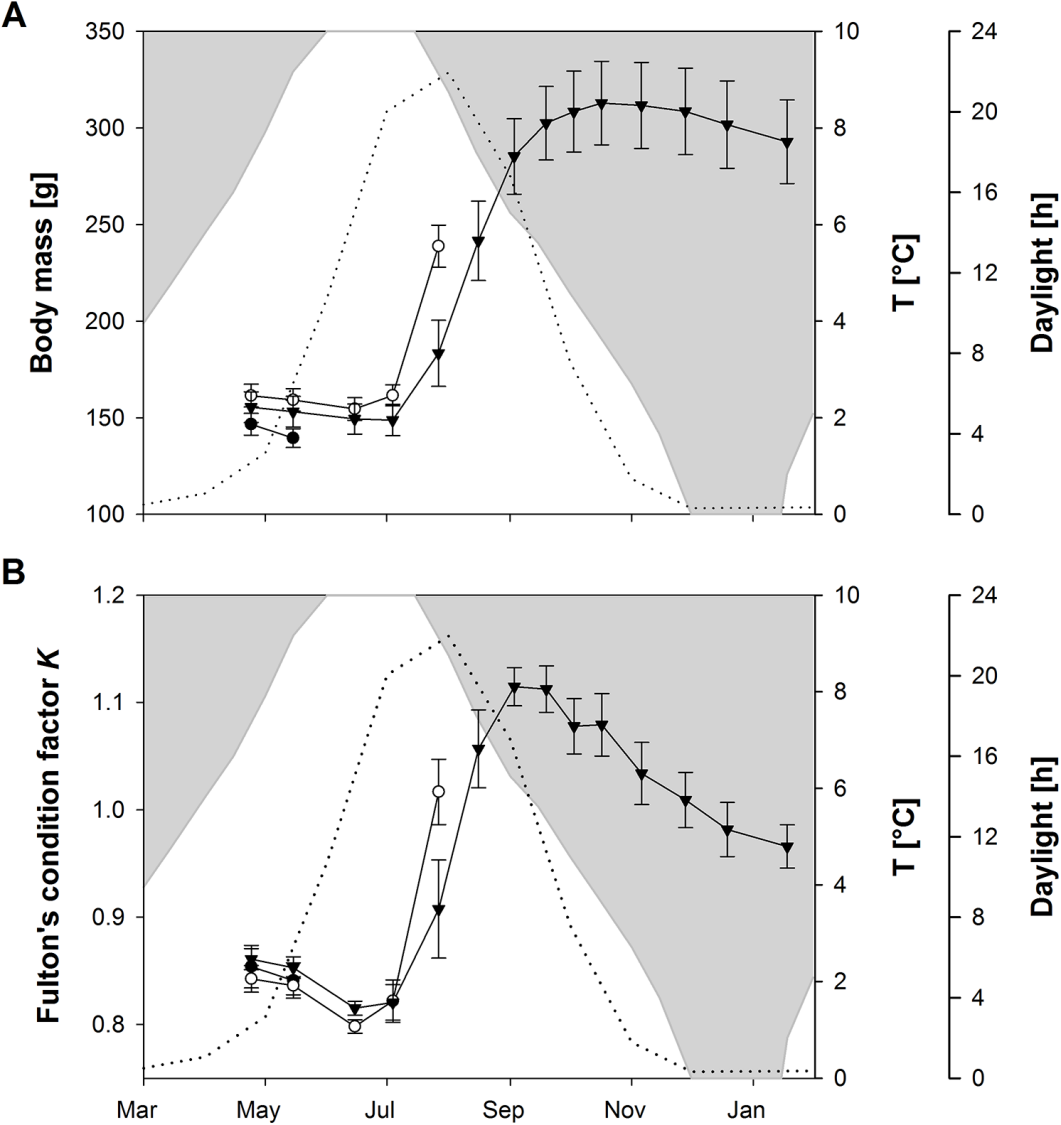
Body mass development (A) and Fulton’s Condition Factor *K* (B) of the fish sampled for mRNA quantification. Black dots: Sampled in May 2012, White dots: Sampled in July 2012, Triangles: Sampled in January 2013. Data are presented as mean (n = 10) ± s.e.m. Dotted line: temperature profile. White area: hours of daylight.

The body mass of the fish sampled on May 15 had decreased between March 28 and the sampling date, indicating that the fish were in a prolonged non-feeding state (Fig 1A). For fish sampled on July 27 there had been a marked increase in body weight from mid-June onwards, indicating that the fish were feeding and growing. Fish sampled on January 18 had gradually decreased in body mass since October, indicating an extended period without feeding. The condition factor *K* (Fig 1B) of the fish sampled in May and January had decreased in the period prior to the samplings, whereas there had been a marked increase in condition factor prior to the sampling of fish in July; this provides confirmation of the feeding status of the sampled fish. All sampled charr were immature and had a *GSI* < 1% (Table 1).

### Relative quantification of gene expression in the three brain compartments

**Fig 2:**
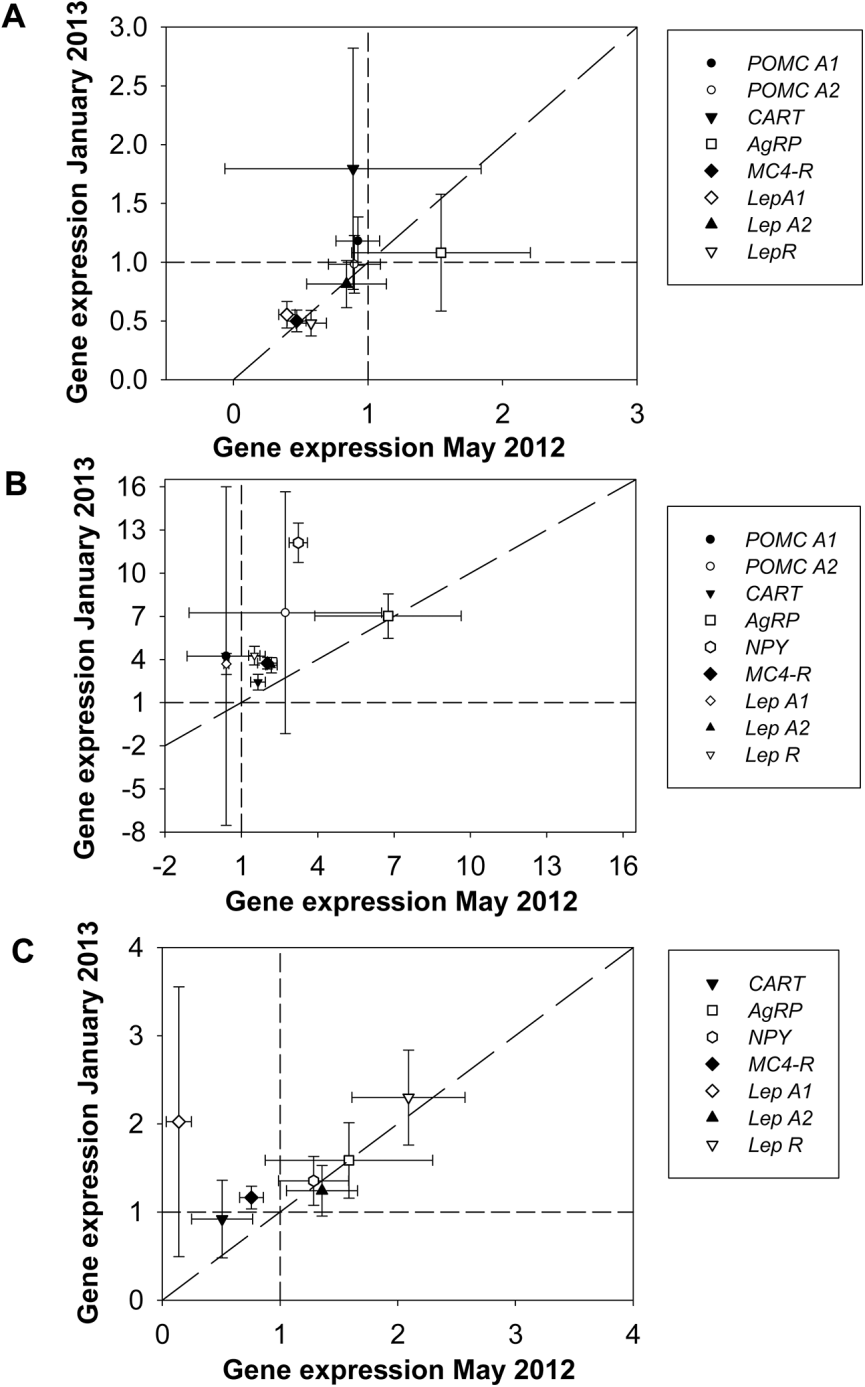
Relative gene expression in the hypothalamus (A), the mesencephalon (B), and the telencephalon (C) in May 2012 and January 2013. Expression levels in brain compartments sampled in May 2012 and January 2013 are presented relative to those sampled in July 2012 (July data were used as calibrator samples and were set equal to one as indicated by the dashed line). Data are presented as mean (n = 8–10) ± s.e.m.

In the hypothalamus there were no significant differences in gene expression of either the anorexigenic *POMC*s, *CART* and *LepA2*, or orexigenic *AgRP* between sampling dates (Fig 2A, Table 3). The expression of *NPY* was very low (C_t_ > cycle 35) on all sampling dates and the data were not analysed statistically. In May and January gene expressions of *LepA1*, *MC4-R* and *LepR* were two-fold lower than in July, whereas no significant differences in the expression of these genes were found between the fish sampled in May and January (Fig 2A, Table 3).

**Table 3:** Testing for differences across sampling time points (p-values for ANOVA and Tukey’s Honestly-Significant-Test).

Gene	Hypothalamus				Mesencephalon				Telencephalon			
	ANOVA	Ma-Ju	Ju- Ja	Ma-Ja	ANOVA	Ma-Ju	Ju—Ja	Ma-Ja	ANOVA	Ma-Ju	Ju-Ja	Ma-Ja
***POMCA1***	n.s	n.s.	n.s.	n.s	n.s.	n.s.	n.s.	n.s.	n.d.	n.d.	n.d.	n.d.
***POMCA2***	n.s.	n.s.	n.s.	n.s.	n.s.	n.s.	n.s.	n.s.	n.d.	n.d.	n.d.	n.d.
***CART***	n.s.	n.s.	n.s.	n.s.	0.001	n.s.	0.001	n.s.	n.s.	n.s.	n.s.	n.s.
***MC4-R***	<0.001	<0.001	<0.001	n.s.	<0.001	0.01	<0.001	0.001	0.011	n.s.	n.s.	0.008
***LepA1***	<0.001	<0.001	<0.004	n.s.	<0.001	n.s.	<0.001	<0.001	0.017	n.s.	n.s.	0.018
***LepA2***	n.s.	n.s.	n.s.	n.s.	<0.001	0.015	<0.001	0.001	n.s.	n.s.	n.s.	n.s.
***LepR***	0.004	0.015	0.006	n.s.	<0.001	n.s.	<0.001	<0.001	<0.001	0.002	<0.001	n.s.
***AgRP***	n.s.	n.s.	n.s.	n.s.	0.004	0.005	0.022	n.s.	n.s.	n.s.	n.s.	n.s.
***NPY***	n.d.	n.d.	n.d.	n.d.	<0.001	0.010	<0.001	<0.001	n.s.	n.s.	n.s	n.s.

Ma—Ju = May tested against July, Ju—Ja = July tested against January, Ma—Ja = May tested against January. n.d.: no data (due to low expression level and/or poor melt curve). n.s.: not significant

In the mesencephalon, relative gene expression of *POMCA1* and *POMCA2* did not differ between the sampling dates (Fig 2B, Table 3). Gene expression of *NPY* was twelve-fold higher in January than in July, and about three-fold higher in May than in July. Expression of *AgRP* was seven-fold higher in May and January than in July (Fig 2B, Table 3). Gene expression of *CART* was 2.5-fold higher in January than in July. No significant differences were found when comparing the expression of *CART* in May and July, and January and May (Fig 2B, Table 3). *LepR* expression was four-fold higher in January than in both May and July, but there was no significant difference between May and July (Fig 2B, Table 3). The expression of *LepA1* was three-fold higher in January than in July and seven-fold higher in January than in May, but no significant differences for *LepA1* expression were detected between the May and July samples. The expression of both *MC4-R* and *LepA2* was three-fold higher in January than in July and 2.5-fold higher in May than in July (Fig 2B, Table 3).

In the telencephalon, there were no significant differences in gene expression between sampling times for the orexigenic neuropeptides *AgRP* and *NPY*, or for the anorexigenic neuropeptides *CART* and *LepA2* (Fig 2C, Table 3). The expression of *LepR* was two-fold higher in both May and January than in July. Expression of *MC4-R* was slightly higher in January than in May, but no significant differences in expression were found between January and July, and May and July (Fig 2C, Table 3).

## Discussion

We aimed to use the seasonal feeding cycle of anadromous Arctic charr to reveal central mechanisms involved in long-term regulation of appetite and energy homeostasis in fish. The temporal changes in body weight and condition factor (Fig 1A and 1B) correspond with those seen in previous studies [24, 41, 46, 47] and confirm the existence of a pronounced seasonality in appetite and growth of Arctic charr when given continuous access to food. The fact that the charr sampled in May and January had lost weight in the period before sampling and had empty stomachs support the existence of a winter non-feeding state, and that they represented individuals from the large fraction of the anadromous charr population that do not feed during the winter [41].

Although the summer feeding period partly coincided with increased water temperature, the most pronounced increases in appetite and growth of the Arctic charr occurred several weeks after water temperature had started to increase, and growth persisted during the period of decreasing water temperature in late summer and autumn (Fig 1). Even though temperature has marked effects on food intake in fish and other ectothermic animals [48], data from the present, and previous studies [41, 49], provide evidence that water temperature is not the determinant of feeding and non-feeding state in charr. Further, changes in photoperiod probably did not trigger the switch from the non-feeding to the feeding state, because photoperiod was increasing during the early part of the experiment, and there were 24 hours of daylight by mid-May when fish were still decreasing in weight (Fig 1). Similarly, previous work has provided evidence that charr display seasonality in food intake independent of changes in photoperiod [50].

It can therefore be concluded that the transitions between non-feeding and feeding states in Arctic charr are physiologically regulated on a seasonal basis and, as such, the Arctic charr could represent an excellent model for investigating mechanisms regulating long-term appetite in fish.

### Differences in relative mRNA abundance of appetite regulators in the brain do not appear to reflect feeding status

If the brain neuropeptides investigated in the present study were regulating long-term appetite, one would expect to find genes encoding for orexigenic neuropeptides to be more highly expressed in feeding charr, sampled in summer, than in non-feeding charr, sampled in spring and winter, and genes encoding for anorexigenic neuropeptides to have lower expression in feeding charr than in non-feeding charr. These expectations were not met.

In the hypothalamus, the expression of neither the anorexigenic *POMC* and *CART* nor the orexigenic *AgRP* genes differed between sampling dates (Fig 2A, Table 3) whereas the gene expression of *NPY* was undetectable with RT-qPCR. These findings suggest that seasonal changes in appetite in anadromous charr are independent of gene expression occurring within the hypothalamic *POMC*/*CART* and *NPY*/*AgRP* system. Furthermore, in the mesencephalon expression of orexigenic *NPY* and *AgRP* was higher in non-feeding charr sampled in January and May than in feeding charr sampled in July (Fig 2B, Table 3). In the telencephalon, the only temporal difference in gene expression was seen for *MC4-R* (Fig 2C, Table 3), but data were not consistent with a direct involvement in seasonal regulation of appetite. Consequently, our findings do not provide support for a cause-and-effect role of these neuropeptides in the seasonal shifts of feeding in the charr.

Few studies have reported how the gene expression of central appetite regulators varies with seasonal feeding cycles in fish. Cunner (*Tautogolabrus adspersus*) had lower expressions of hypothalamic and telencephalonic *NPY* and *CART* during winter torpor, when they voluntarily fast, than during their summer feeding season [51]. In the winter flounder (*Pseudopleuronectes americanus*), on the other hand, hypothalamic gene expression of *NPY* was higher in winter, when food intake was low, than in summer, when food intake was high, whereas the expression of *CART* did not differ across seasons [52]. Thus, available data do not provide a clear picture about how central appetite regulators participate in the regulation of seasonal feeding cycles in fish, or if they are involved at all. In seasonal mammals, e.g. Siberian hamster (*Phodopus sungorus*), seasonal changes in appetite and body weight may not be accompanied by seasonal changes in the expression of central appetite regulators, suggesting that the central appetite regulating system predominantly regulates short-term food intake by timing of meals and reactive responses to changes in food availability [53]. This assumption is in accordance with observations that, in contrast to animals that are subjected to feed deprivation, the mechanisms that normally defend body mass and/or energy status are shut down during periods of body weight change in seasonal mammals [54]. Such a mechanism, rooted in models which propose a seasonal sliding set-point in adiposity [55], would imply that a decrease in body mass and adiposity during winter would not induce a re-commencement of feeding. This might explain the observation made during the present (Fig 1, Table 1) and previous [41] studies on Arctic charr.

### Seasonal differences in central gene expression of *Lep* and its receptor point towards involvement in seasonally regulated processes

Lep plays an important role in maintaining energy homeostasis in mammals by signalling adiposity to the brain, but, hitherto, no relationship has been found between plasma Lep levels and adiposity in fish [24, 27, 29, 56]. In anadromous Arctic charr plasma Lep concentrations did not change significantly during the annual feeding cycle, despite there being marked differences in body fat that ranged from 4.5% in early summer to 17% in autumn [24]. In fish, *Lep* and *LepR* are expressed in several tissues including the brain [28, 32]. Hence, our study aimed to investigate whether centrally expressed *Lep* and *LepR* could be linked to long-term appetite regulation in charr.

To the best of our knowledge, this is the first study in which central expression of *Lep* and *LepR* has been analysed through a seasonal feeding cycle in fish, including voluntarily non-feeding periods. Our results do not indicate that centrally expressed *Lep* acts as an anorexigenic agent in a long-term perspective, because the hypothalamic gene expression of *LepA1* and *LepR* was higher in feeding charr than in non-feeding charr, and the expression of *LepA2* did not differ between feeding and non-feeding charr (Fig 2A).

Nevertheless, a role for central LepR in appetite regulation is possible, because recombinant Lep treatment reduced feeding in both rainbow trout (*Oncorhynchus mykiss*) [23] and Atlantic salmon [57]. In both studies, the anorexigenic effect of Lep was associated with an upregulation of the hypothalamic [23] and brain [57] POMC system, which in mammals promotes Lep-mediated anorexigenic effects through the MCR pathway [1, 2]. In charr, there was not a consistent seasonal pattern in the expression of *LepR* and *POMC A1* and *A2*, but there was a covariation of expression of *LepR* and *MC4-R* in the hypothalamus (Fig 2A).

In the mesencephalon of the Arctic charr *LepA2* expression was, in contrast to the hypothalamus, higher in non-feeding fish sampled in January and May than in feeding fish sampled in July, while *LepA1* expression was higher in fish sampled in January than in fish sampled in July (Fig 2B). Whether Lep produced in the mesencephalon acts as an anorexigenic agent in non-feeding charr during winter and spring requires further investigation.

Also there is a need to confirm if differing expression patterns of *Lep* and *LepR* across brain compartments are indicative of autocrine and paracrine signalling, as has been suggested previously [47, 58].

It is possible that changes in hypothalamic *LepR* expression (Fig 2A) could be related to seasonal shifts between anabolic and catabolic states and/or to body adiposity. In support of this conjecture, *LepR* was more highly expressed in the brain of juvenile Atlantic salmon fed to satiety than in conspecifics subjected to feed restriction [56]. On the other hand, central *LepR* expression was negatively correlated with body fat in juvenile Atlantic salmon sampled during the growth season [59]. These discrepancies may be related to the fact that both studies monitored *LepR* transcripts in the whole brain. The different seasonal patterns in *Lep* and *LepR* expression in different brain compartments in the present study (Fig 2) emphasise the need for conducting analyses in separate brain regions or even at the level of specific nuclei.

Overall, the seasonal patterns of central expression of *Lep* and *LepR* and the differences between the three brain compartments support the view that Lep exerts pleiotropic effects in fish [60]. Seasonal differences in central expression of *Leps* and *LepR* may be linked to one or more of the physiological events that characterize the seasonal life of anadromous Arctic charr. These encompass substantial changes in feeding, substrate utilization and energy expenditure [37], a vernal smoltification preceding seaward migration [61] and seasonal reproduction [62].

## Conclusions

This study did not reveal a link between expression of anorexigenic (*CART*, *POMC*s and *MC4-R*) and orexigenic (*NPY* and *AgRP*) neuropeptides in different brain compartments and seasonal changes in appetite, growth and condition in Arctic charr. Nor did we find support for an anorexigenic role of centrally expressed *Lep* in the long-term regulation of appetite in charr. Some words of caution must, however, be given because changes or lack of changes in mRNA abundance do not necessarily reflect changes in their encoded peptides, as post-transcriptional regulation may occur [29].

Some temporal variations in gene expression were observed, and there were differences across brain compartments. Consequently, our results indicate the need for additional studies to unravel the roles of these neuropeptides in governing physiological functions in fish.
